# Enhancing professional success: Chinese EFL teachers’ workplace buoyancy and cognitive flexibility

**DOI:** 10.1016/j.heliyon.2023.e13394

**Published:** 2023-02-04

**Authors:** Shengji Li

**Affiliations:** School of International Education, North China University of Water Resources and Electric Power, Zhengzhou, Henan Province, PR China

**Keywords:** EFL learners' achievement, Intellectual development, EFL teachers, Professional success, Buoyancy, Cognitive flexibility

## Abstract

As integral to the EFL learners' achievement and intellectual development, good language teachers need to be familiar with the influential factors hindering or promoting their success. Positive psychology paying attention to positive traits highlights the factors that boost performance. In this line, by taking boosters of being a successful teacher, the aim of the current study is to consider the interplay between Chinese EFL instructors' professional success, workplace buoyancy, and cognitive flexibility. Then, it is considered whether EFL teachers' buoyancy and cognitive flexibility can predict success. Accordingly, 328 Chinese EFL teachers were conveniently selected to participate in this study. To analyze the collected data, multiple regression analysis and Spearman Rho correlation index as well as ANOVA were employed. The findings are an indication of a direct and significant correlation and coaction among EFL teachers’ cognitive flexibility, workplace buoyancy, and professional success. Additionally, the results indicate that the cognitive flexibility of EFL teachers can be one of the significant predictors of success in their profession. Our findings imply that teachers need to enhance their positive personality traits such as cognitive flexibility and buoyancy to be successful in their profession.

## 1 Introduction

Widely believing in the critical role of the teachers in educational systems makes it indispensable to enhance their ability to think and screen their performance and behavior in the classroom. In other words, teachers should be able to monitor and manage their own thoughts as well as their teaching in classroom. Conceiving language educators as a driving force of change, Brown [1] posits that language teachers have the capability to psychologically cooperate instead of competing, to empower their weaknesses, to resolve the problem rather than declining, and to understand the contextual issues instead of being prejudiced. As a multifaceted activity, teaching is widely influenced by teachers' quality and their personality traits [2]. The teacher's quality and characteristics are determined by several factors including thinking skills, affective, cognitive, and personality traits. Brown [3] believes that teachers' emotions, personality traits, and other affective variables are contributing factors to being a successful teacher. Exploring and understanding these factors and enhancing the constructive ones seem necessary given the recent changes in the education world and consequent pressure on teachers. We assume that teachers' workplace buoyancy and their cognitive flexibility, as two personal capacities, can enhance EFL teachers' professional success in the challenging context of EFL.

Teaching is not easy, and it has become additionally challenging and burdensome over the past decades due to changes called “intensification” [4]. “Intensification”, looking at education from an economic-driven perspective, has led teachers to be engaged in imposed administrative tasks from high-stake holders besides their teaching-related responsibilities. This type of workload, which is unrelated to teaching action, is transferred into teachers' every-day-experience of work [5]. Day and Gu [6] argue that the dominance of everyday challenges and hardships encountered by teachers emphasizes the significance of having “everyday resilience” (p. 210), i.e., buoyancy to make them able to teach to the best. To avoid being overwhelmed, teachers need to rebound from continuous challenges quickly. Otherwise, they will face the risk of being suffered from more severe emotional problems leading to a decline in their work [7]. Martin and Marsh [8] define buoyancy as an individual's aptitude to effectively cope with low-level and everyday hardships. Researchers have stressed the relevance of buoyancy to situations with difficulties including educational contexts and its salience in different populations entailing teachers [9]; although, to date, the role that buoyancy plays in teachers' performance and success has remained unexplored.

The other factor which is assumed to be important in EFL teachers' success is related to teachers' cognitive flexibility. Stronge [10] believes that teachers need to be planned along with being spontaneous and flexible when they are in demand of being responsive. This is one of the teacher characteristics which directly influence effective teaching. Considering the inherent dynamicity of the language teaching process, language teachers and learners may encounter some unanticipated events and situations which can hinder or facilitate teaching and learning. Teimourtash [11] believes that teachers need to be aware of these situations and be flexible to immediately control these unpredictable situations and manage them appropriately by adaptable choices to reach the right goal. Park [12] argues that to be a flexible person in different social and educational contexts, every person should be cognitively flexible. Cognitive flexibility (CF) is an individual's ability to instinctively rearrange his/her knowledge responding adaptively to complete change of situational requirements. This competency is the result of knowledge representation manner and the operation process of these mental representations [13]. Teachers' flexibility, which is related to their awareness and their readiness to adaptable performance, is applicable to all teaching contexts including EFL one [11].

In the EFL context, with the well-known characteristic of being occupied by challenges and adversities, EFL educators must frequently tackle several socio-economic hardships and dejections along with unpredicted situations which constantly slow their engagement and performance [14]. Following the positive psychology and its emphasis on the effects of positive personality traits on individuals' successful performance [15], we assumed that the buoyancy and cognitive flexibility can be critically important in order to be strong and positive in the face of the inherent daily challenges of L2 education, and consequently, professionally successful. To our knowledge, no study has been done to understand these variables concerning EFL teachers, their impacts, and their predictive power on EFL teachers' success. To fulfill the demand for more study, we make an effort to explore the contribution of teachers' buoyancy and cognitive flexibility to ELF instructors’ professional success. Therefore, two related questions are generated:

1. Are there any meaningful coaction among workplace buoyancy, cognitive flexibility, and professional success of Chinese EFL teachers?
2. Can workplace buoyancy and cognitive flexibility of Chinese EFL teachers be significant predictors of success in their profession?

## 2 Literature review

### 2.1 Teachers’ success

Given teachers' principal role in education, teachers' success is one of the noticeable realms in the pedagogical research and foreign language teaching/learning context [[16], [17], [18]]. Hung, Oi, Chee, and Man [19] believe teachers' success is a sense of success originated from their work. More soundly, teachers' achievement may entail acquiring content knowledge, upgrading their teaching techniques, learning skills to improve behavior patterns concerning relationships and interactions with their students, and getting promotions, to name a few. Brookfield [20] characterizes a successful teacher as teaching at an appropriate rate and pace, taking advantage of several instructional strategies, checking learners' comprehension and participation, paying attention to educational objectives and syllabus along with having a sense of humor. Anderson [21] assumes that successful teachers define their goals and try to gain the necessary knowledge and skills to accomplish their intentions. Korthagen [22], to present “the essence” of a successful teacher, considers these layers: Belief, Competency, Identity, Behavior, and Mission. The operation of these five layers may be influenced by different circumstances, i.e., Environment. The Belief layer is relevant to ‘What do I think?’; Competency answers ‘What am I capable of?’; Identity deals with “Who am I in my work?”; Mission is related to ‘What inspires me?’; Behavior answers ‘What do I carry out?’; and finally, Environment is associated with ‘What am I dealing with?’

Over the literature, different questionnaires were developed and validated to rigorously assess teachers' success [23]. Dordinejad and Porghoveh [24] argue that besides having area knowledge, teachers need to evaluate their motivation, beliefs, and self-regulatory factors. Another line of studies directs their focus on the contribution of several factors to teachers' success. Hung et al. [19] find teachers' concerns, attitudes, and expectations in correlation with teachers' success. In Duckworth, Quinn, and Seligman's studies [25], life satisfaction, grit, and optimistic explanatory style predict teachers' success. Pishgadam, Derakhshan, Zhaleh, and Al-Obaydi [26] find a positive correlation between teachers' stroke success. Derakhshan, Coombe, Zhaleh, and Tabatabaeian [27] find out the significance of EFL teachers' interest in inquiry and occupational development regarding success. Hargreaves [28] emphasizes the importance of teachers' emotions and teacher-student relationships. Moreover, Derakhshan et al. [27] focus on the role of teachers' identity and autonomy, which directly and positively correlates with teachers' success. In the EFL context, teachers' critical thinking ability is reported to be in positive correlation with teachers' professional success [29]. Malmir [30] examines the predictive power of reflective teaching and self-efficacy in relation to teachers' professional success in the Iranian EFL context. Partovi and Tafazoli [31] stipulate that EFL teachers' occupational achievement is defined by various qualities such as resilience, self-regulation, and teaching background.

Overall, considering the elaborateness and challenges of teaching, gaining success in all teaching contexts cannot be achievable; consequently, identifying the influential factors which facilitate or hinder teachers' success seems to be necessary [17,19]. The reviewed evidence insinuates additional research is needed on contributing factors to EFL teachers’ success. This scanty requires more work to fill this lacuna. In this line, we aim to look at the interplay among cognitive flexibility, workplace buoyancy, and professional success of Chinese EFL teachers as well as the predictive power of these factors with regard to success.

### 2.2 Buoyancy

Several pieces of research have been done on the relationship between teachers' engagement, achievement, and resilience [[32], [33], [34]]. Recently, however, psychologists argue that resilience in its traditional definitions is based on chronic life challenges and difficulties like trauma [35], or acute stress [36] such as economic loss, violence, or learning disabilities. These untypical adversities do not occur frequently. Therefore, the recent studies have been extended to focus on “everyday” resilience since the challenges faced by teachers are daily hassles like students' emotional problems or lack of classroom resources [37]. This daily resilience has been known as buoyancy. Buoyancy is individuals’ ability to successfully resolve daily challenges and pass holdups of everyday life like difficult tasks and competing deadlines [8]. In order to measure academic Buoyancy, Martin and Marsh [8,38] develop two Academic Buoyancy Scales (ABS), one for students and the other for school personnel including teachers called Workplace Buoyancy Scale (WBS).

Stemming from positive psychology, academic buoyancy emphasizes the position of feeling and emotions in education [39,40]. As a psychological construct, academic buoyancy reflects the ordinary academic obstacles and impediments in a constructive setting [41]. It concerns an individual's competency to diagnose and manage challenges that arise in his/her academic life. Academic buoyancy, in contrast to resilience, is a productive, positive, and flexible response focusing on everyday academic setbacks [42,43]. As with most of the other constructs in positive psychology, academic buoyancy is under the effect of contextual issues (external factors) and personality variables and traits (internal factors) [44,45]. Martin and Marsh [46] highlight academic buoyancy benefits more from “strengths”, exploit proactive approaches in challenging situations, and finally try to ignore “extreme cases” and explore “many and healthy” cases. On account of this positive orientation toward emotions, Zhang [47] holds that academic buoyancy is considered the “positive” type of resilience. In this line, being grounded in the differentiation between buoyancy and resilience, the “teachers' buoyancy” construct is proposed with the aim of concentrating on teachers' recovery manner from daily repetitive setbacks in the teaching world. Operationally, buoyancy is a particular self-perceived adaptive reaction to hardships and obstacles [8]. Accordingly, teachers' buoyancy refers to their self-perceived adaptive reaction to typical setbacks and hardships of everyday teaching [48].

As with other educational contexts, academic buoyancy gains increasing attention in the EFL context due to devastating setbacks and adversities natural to L2 education. Therefore, to be able to operate acceptably in the EFL contexts, an individual is in needs to identify the difficulties and develop appropriate coping strategies [49]. Researches on the positive effects of buoyancy on students' success, participation, decreasing stress, competence, self-regulation, and exam performance have been carried out around the world [41,43,50]. However, teachers’ buoyancy remains unexplored. Therefore, there is a need to explore this area deeply among both students and educators.

### 2.3 Cognitive flexibility

Despite several studies on cognitive flexibility (CF) and increasing interest in the learning context, there is no consensus on the definition and measurement of cognitive flexibility. One generally accepted operational definition of CF is the ability to shift one's cognitive tasks to make them suitable for the varying conditions [51]. Cognitive psychologists define CF as the capability to successfully adjust to circumstances by switching over different brain works, or it is the potential of facing different difficulties using multidimensional strategies [52]. Mentioning other terms used for CF such as cognitive shifting or mental set, task shifting, Carvalho and Moreire [53] explain cognitive flexibility in terms of moving from old thoughts to new ones depending on the requirements of the new situations including multiple dimensions of thought simultaneously. Deak [54] discusses that cognitive flexibility is self-motivated adjustment, formation, and initiation of cognitive processes on the basis of data from environment, both linguistically and non-linguistically, in response to varying and unstable task requirements. As contextual factors changes, the cognitive system has the capability of adjusting to new conditions through attention shifting, relevant data selection to choose future responses, planning, and constructing new initiation conditions to react to the situation [55]. Likewise, according to Elen, Stahl, Bromme, and Clarebout [56], an individual's ability to consider his or her choices mindfully and in balance is known as CF. They point out that CF is noticeable in one's adaptation to situational shifts, findings resources and choices, searching for new knowledge, being ready to change, tentatively working with ideas, and adapting to new situations. Therefore, individuals with cognitive flexibility think automatically, deeply, and flexibly instead of looking at situations mindlessly.

The notion of cognitive flexibility has been investigated since the 1990s and is explicated as an individual's mindfulness of her/his choices being suitable for recent situations, conforming to new settings, and being ready to act flexibly [57]. Several studies have shown that people with higher levels of CF can successfully cope with stress and anxiety [58], can effortlessly get used to new and current circumstances [59], and can manage their anger [60]. Researchers have also explored CF and its role in the educational context. For example, Edmunds [61] studies the association between teachers' sense of self-efficacy and their CF. In another study, a negative correlation between CF, depression, and anxiety is found in teacher candidates [62]. His findings indicate individuals' worries decrease as their flexibility increases.

Cognitive flexibility, as with general education, has been studied in the EFL context. However, they are few. Deak [54] maintains that cognitive flexibility is required in EFL contexts since most EFL teachers and learners sometimes face unpredictable linguistic situations or cultural driven problems [63], in which CF is required to deal with raised problems. Kılıç and Demir [64] study teachers' perceptions of teaching-learning environments grounded in cognitive flexibility. The outcome of this study was teacher candidates' positive attitudes toward such classes. In another study, Saffarin and Fatemi [55] explore the relationship between EFL instructors' CF and pupils' perspectives toward learning English. The results show a positive relationship and the necessity of adding CF into foreign language teaching. In line with these studies and with the hope of contributing to broadening the literature on the effects of CF in EFL education, this work tries to examine the coaction between EFL teachers’ CF and professional success.

## 3 Methodology

### 3.1 Participants

The participants comprise 328 EFL teachers (valid cases) from mostly Jiangsu province in China. The selection of teachers is done by applying convenience sampling from adult English teachers, male and female, with different English language proficiency levels aged 20–60. Consent is given to all participants before they are selected via Wenjuanxing (https://www.wjx.cn/), online data-collection software in China. Since the data are collected mostly from one province (317 = 96.064%), the generalizability of the findings should be done with caution.

### 3.2 Instruments

For this study, the data collection is done by exploiting a Likert scale, with four sections including demographic information, teachers' CF scale, teacher success scale, and workplace buoyancy scale. The CF scale is the *Cognitive Flexibility Scale* [57]; the workplace buoyancy questionnaire is taken from *Buoyancy Scale* [38]; finally, the teacher success scale is adopted from the *Characteristics of Successful Language Teachers Questionnaire (CSLTQ).* This scale is created and examined in terms of its validity by Babaii and Sadeghi [65] to assess teachers' insights of a successful language teacher attributes. In this study, The teacher Cognitive Flexibility Scale with 0.89 reliability consists of 12 Likert items, representing participants' agreement or disagreement with six points ranging from 1: Strongly disagree to 6: Strongly agree. The CSLTQ, with 0.93 reliability, covers eight features in 46 items, ranging from 1: Strongly Disagree to 5: Strongly Agree. Finally, the questionnaire on teachers' workplace buoyancy with four items and 7 points (ranging from 1: Strongly disagree to 7: Strongly agree), and 0.90 reliability is employed to measure participants’ opinions on their buoyancy.

### 3.3 Data collection procedure

Because all participated teachers are form China, to make the questionnaire fully expressed and easily understood, the researcher in cooperation with several experts in English translation and applied linguistics have translated the questionnaires into the Chinese language. The Chinese versions have been double-checked by experts for their clarity and correctness before being distributed online by Wenjuanxing (https://www.wjx.cn/) via WeChat or other electronic devices. Since the researcher makes no acquaintance with any one of the participants, there is no manipulation in any form of the data or any interest conflict between the researcher and the participants. All participants are ensured of the confidentiality of their information and responses and their use only for research purposes. They are also guided on the mechanism of filling in the questionnaire using electronic devices and the way of dealing with any potential problem may be encountered. Altogether, 328 questionnaires (all valid) are collected. They are carefully examined before being launched to SPSS software. Finally, the processed data is used to answer the research questions (see Table 1).

## 4 Results

To confirm the reliability of the employed questionnaires, a Cronbach Alpha is administered. Table 2 presents the satisfactory reliability of all three instruments including teachers’ success (0.98), workplace buoyancy (0.90), and cognitive flexibility (0.89) with satisfactory Cronbach Alpha indices.

**Table 1:** Demographic information of participants.

Demographic Information Category	*N*	*%*
**Education Level**		
Associate of Arts	289	88.1
Bachelor of Arts	29	8.84
Master of Arts	10	3.04
Ph.D.	328	100
Total	328	100
**Gender**		
Male	54	16.46
Female	274	85.54
Total	328	100
**Major**		
English language literature		9.08
Applied linguistics		5.21
Teaching English as a foreign language		73.48
English language translation		12.23
Total	328	100
**Teaching Experience (year)**		
1–15		25.68
16–30		35.57
31–45		18.21
46–60		20.54
Total	328	100

**Table 2:** Instrument reliability.

Questionnaires	Cronbach's Alpha		Item Numbers
Teachers' success	.98		46
Workplace Buoyancy	.90		4
Cognitive Flexibility	.89		12

After ensuring the reliability of the instruments, the normality of the data is checked to decide on its parametric or non-parametric analysis. As Table 3 illustrates *P* values are less than the significance level (0.000) as for all of the variables indicating the non-normality of the data obtained from any of them. Thus, they violate the normality assumption and the data analysis required to exploit a non-parametric approach. Therefore, a Spearman Rho correlation index is employed.

**Table 3:** Test of normality.

	Kolmogorov-Smirnov^a^		
	Statistic	df	Sig.
Teachers' success	.123	337	.000
Workplace Buoyancy	.227	337	.000
Cognitive Flexibility	.140	337	.000

### 4.1 The first RQ

RQ1 Are there any meaningful coaction among workplace buoyancy, cognitive flexibility, and professional success of Chinese EFL teachers?

Spearman correlation index presents the direction and the magnitude of the relationship among the workplace buoyancy, CF, and teacher success. The directness of the relationship among the variables is demonstrated in Table 4, i.e. the higher amount of one shows the greater level of others. Additionally, all these relationships benefit from the significance level of .000. It suggests a meaningful association among the variables.

**Table 4:** Correlation among teachers’ success, workplace buoyancy, and cognitive flexibility.

			TS	TWB	TCF
Spearman's rho	TS	Correlation Coefficient	1.000	.578^a^	.698^a^
		Sig. (2-tailed)	.	.000	.000
		N	337	337	337
	TWB	Correlation Coefficient	.578^a^	1.000	.695^a^
		Sig. (2-tailed)	.000	.	.000
		N	337	337	337
	TCF	Correlation Coefficient	.698^a^	.695^a^	1.000
		Sig. (2-tailed)	.000	.000	.
		N	337	337	337

a Correlation is significant at the 0.01 level (2-tailed).

### 4.2 The second RQ

To answer the second research question, which concerns if workplace buoyancy and teachers’ cognitive flexibility of Chinese EFL teachers can be significant predictors of success in their profession, a multiple regression analysis is run and the subsequent Tables are produced:

The magnitude of the variance in the scores of the teachers' success (dependent variable) justifiable by the model (entailing cognitive flexibility and workplace buoyancy) is disclosed in Table 5. In this case, the value was 0.75 (*R*^*2*^ = 0.57). Articulating in percentage, the model (covering scores of cognitive flexibility and workplace buoyancy) accounts for 57% of the variance of scores obtained from teachers’ success.

**Table 5:** The Relationship model among Teachers’ Success, Workplace Buoyancy, and Cognitive Flexibility.

Model	R	R Square	Adjusted R Square	Std. Error of the Estimate
1	.756	.571	.568	15.39
a. Predictors: (Constant), Teachers' Cognitive Flexibility and Workplace Buoyancy				

To examine the results’ significance statistically, it is indispensable to check Table 6, ANOVA. Table 6 assesses the assumption that multiple R in the populace be the same as zero (0). The statistical significance is reached by model (*F* = (2, 334) = 222.17, Sig = 0.000, this actually suggests p < .05).

**Table 6:** The relationship among success, workplace buoyancy, and cognitive flexibility, ANOVA.

Model		Sum of Squares	df	Mean Square	F	Sig.
1	Regression	105364.61	2	52682.30	222.17	.000
	Residual	79197.88	334	237.11		
	Total	184562.50	336			

a. Dependent Variable: Teachers' Success.

b. Predictors: (Constant), Teachers' Cognitive Flexibility and Workplace Buoyancy.

In order to define the more significant variable (cognitive flexibility or workplace buoyancy) in the model which plays role in predicting teachers’ success, the “Beta” in Table 7 is checked. To judge the different variables, looking at the *standardized* coefficients, not the *unstandardized* ones are required. “Standardized” indicates that the values of different variables should have the same scale to be comparable.

**Table 7:** Coefficients for teachers' teachers’ success, workplace buoyancy, and cognitive flexibility.

Model		Unstandardized Coefficients		Standardized Coefficients	t	Sig.
		B	Std. Error	Beta		
1	(Constant)	41.764	7.848		5.321	.000
	Workplace Buoyancy	.634	.377	.087	1.681	.094
	Cognitive Flexibility	2.873	.214	.691	13.406	.000

a. Dependent Variable: Teachers' success.

The present study is done by using beta values to evaluate the involvement of each independent variable. By examining the beta column, it is found that Teachers' cognitive flexibility gains the largest beta coefficient: 0.69. Weigh it against the beta value for the workplace buoyancy, teachers' cognitive flexibility makes the most powerful unique part to justify teachers’ success keeping the explained variance by all other variables under control. It is mentionable that the Beta value for the workplace buoyancy is not significant (sig = 0.09).

## 5 Discussion and conclusion

The current study is essentially conducted to search the role of EFL teachers' cognitive flexibility and workplace buoyancy in their professional achievement. Consequently, it followed two objectives: firstly, to find out whether there is any correlation among the variables of the study i.e. EFL teachers' cognitive flexibility, their buoyancy, and success in their profession. After that, the predictive power of cognitive flexibility and workplace buoyancy in relation to Chinese EFL teachers' success is examined. Reviewing the literature of factors effective in EFL teachers’ success in work, as to our knowledge, the relationship between these three factors has not been inspected. Therefore, we try to find related facts on these new concepts rather than supporting or rejecting previous studies.

The findings for the first research question show the directness of correlation between cognitive flexibility, workplace buoyancy, and EFL teachers' success in their work. To be specific, blessed EFL teachers with a greater capacity of cognitive flexibility have a greater amount of success, and buoyancy reciprocally. The results on the relationship between Chinese EFL instructors’ CF, buoyancy, and success illustrated a significant correlation. To justify this finding, we consider the relationship between cognitive flexibility and success and buoyancy and success separately.

As is known to all, the teaching context, particularly the EFL teaching context, is full of challenges and unpredicted situations influencing their engagement and success. These unexpected situations require teachers to be cognitively flexible to be able to adaptively act in their workplace. In this post-modern era, everything changes quickly and novel approaches and techniques are suggested regularly; therefore, as the members of this modern world, language teachers are in demand to be able to adapt themselves quickly to unanticipated changes to be competent to advance in their teaching profession. Considering Anderson [59] who argues that people benefiting from high levels of cognitive flexibility adapt effortlessly to unexpected situations, it can be also true with EFL teachers. EFL teachers with a higher level of cognitive flexibility can better manage their stress [57], worry less [66], and control their anger [60], all of which lead to emotion regulation and correct decision making. Our finding, on the relationship between EFL instructors’ CF and success, is consistent with the findings of Stahl and Pry [67] who find that individuals with a satisfactory level of CF can efficiently manage challenging and novel circumstances and generate substitute thoughts, and consequently decide correctly.

Regarding the relationship between workplace buoyancy and EFL teachers' success, we find a direct and significant relationship between teachers' buoyancy and success. As Dong et al. [49] argue, “due to the overwhelming adversities and challenges inborn in L2 education”, it is necessary for EFL teachers to be familiar with the hardships of the EFL contexts and adapt proper and required coping strategies in order to be strong confronting adversities [50]. As a positive form of resilience, it can be argued that teachers’ buoyance can be enhanced by constructive connections with knowledgeable individuals by assisting them with gaining fresh thought into alternatives to handle diverse hardships and challenges in the teaching setting.

Given the second research question, the results show that both the variables, cognitive flexibility, and workplace buoyancy, predict the Chinese EFL teachers' success. However, considering them separately, the results indicate that teachers' cognitive flexibility makes the strongest unique contribution to explaining their success. The predictive power of workplace buoyancy is not significant. This finding can be justified, as mentioned above, in the power of being able to create alternative thoughts and pro-activeness which leads to correct decision-making in new and difficult situations. In this regard, Boger-Mehall [68] points out that people's functionality to simultaneously consider a couple of ideas and switch over various dimensions of thought is a vital component of teaching and integral to success.

As mentioned before, teaching particularly EFL teaching is an occupation burdening stress, anxiety, and responsibility on educators. In consequence, in many contexts, teachers feel overwhelmed and leave the job. Acknowledging this, most educational systems admit the worth of teachers’ emotions after the advent of positive psychology. To be successful and fulfill the teaching requirement, teachers should be academically buoyant and cognitively flexible facing setbacks. To survive and accomplish in such stressful contexts, teachers should be prepared and trained to establish necessary skills and caring rapport and engage in the class through applying appropriate strategies.

As it is usual in literature, the present study experiences some limitations. Firstly, the present study is conducted during COVID-19 pandemic precluding researchers from collecting data in person, whereby, the questionnaire is dispersed online. Since, during this pandemic period, teachers teach online, their performance and thoughts may differ from normal time that may influence the results of the study. The next important limitation concerns the findings generalizability. Since the data is collected from EFL instructors mostly in one province of China, the use of findings and generalizability to the other EFL context should be with caution. It is suggested that future studies replicate the data collection in-person and in different EFL contexts with participants from different cultures.

## Author contribution statement

Shengji LI, Ed. D.: Conceived and designed the experiments; Performed the experiments; Analyzed and interpreted the data; Contributed reagents, materials, analysis tools or data; Wrote the paper. </p>

## Funding statement

This work was supported by North China University of Water Resources and Electric Power, “2021 10.13039/501100008554NCWU Education and Teaching Research and Reform Project” [(2022)No. 8], 10.13039/501100002338Department of International Cooperation and Exchange, Ministry of Education. P.R China [(2020) No. 173].

## Declaration of competing interest

The authors declare no competing interests.
