# Supplementary material for: Enhancing professional success: Chinese EFL teachers’ workplace buoyancy and cognitive flexibility
Source: Heliyon. 2023 Feb 4;9(2):e13394. doi: 10.1016/j.heliyon.2023.e13394 (PMC9929303; doi:10.1016/j.heliyon.2023.e13394)
Supplement: Multimedia component 1 [file mmc1.docx]

**Supplementary File**

**Enhancing Chinese EFL Teachers’ Professional Success: The Role of Teachers’ Workplace Buoyancy and Cognitive Flexibility**

**Giving Consent**

I hereby declare that I voluntarily participated in this study. I let the researchers use my responses as data as far as my identity remains anonymous. In addition, the researchers guarantee that all information I provide for this study will be treated confidentially.

Yes

No

**Demographic Information**

**Gender:**

Male

Female

**Age:**

**Major:**

Applied Linguistics

Linguistics

English Language Literature

English Language Translation

Teaching English to Speakers of Other Languages (TESOL)

Teaching English as a Foreign Language (TEFL)

Teaching English as a Second Language (TESL)

Other

**Last academic degree obtained:**

Diploma

Associate of Arts

Bachelor of Arts

Master of Arts

Ph.D.

Other

**Country where you are currently teaching:**

**Teaching experience:**

1-5

6-10

11-15

16-20

21-25

26-30

31-35

36-40

41-45

46-50

51-55

56-60

**Level of education you are currently teaching:**

**Questionnaires:**

**Part One. Teacher Success**

Instruction: Please read the following list carefully. For each statement, select the response that best represents your Agreement or Disagreement. Ranging from 1 (Strongly disagree) to 5 (Strongly agree).

**An effective English teacher is someone who should:**

1. Understand spoken English well.

2. Know English culture well.

3. Read English well.

4. Have a high level of proficiency with English vocabulary.

5. Write English well.

6. Pronounce English well.

7. Speak English well.

8. Be fully familiar with English grammar.

9. Prepare the lesson well.

10. Follow syllabus tightly.

11. Use particular methods and techniques in teaching.

12. Manage the class time well.

13. Assign homework.

14. Integrate group activities to class.

15. Be up-to-date (e.g. use internet and recent technologies in teaching).

16. Teach how to learn English outside the classroom (teach language learning strategies).

17. Use lesson plans.

18. Teach English adapted to students’ English proficiency levels.

19. Maintain good classroom atmosphere using authority, if necessary.

20. Teach English in English

21. Teach English in Persian (students’ native language)

22. Assess what students have learned reasonably.

23. Provide opportunities to use English through meaningful tasks and activities.

24. Provide activities that arouse student’s interest in learning English.

25. Be helpful to students in and outside the classroom.

26. Be available for students.

27. Alleviate students’ anxiety in English class.

28. Listen to student’s opinions and let them express themselves.

29. Help students to develop self-confidence in order to learn English well.

30. Be friendly to students.

31. Have a good sense of humor.

32. Not discriminate between students and treat them fairly.

33. Arouse students’ motivation for learning English.

34. Show interest in students (by remembering students’ names) and their learning.

35. Be neat and tidy in appearance.

36. Pay attention to the personal needs of students.

37. Be polite and respect the personality of the students.

38. Be disciplined.

39. Be punctual.

40. Be open to criticism.

41. Be flexible.

42. Be attentive in the class.

43. Be interested in his/her career.

44. Not lose temper and get angry.

45. Stick to administrative rules and regulations.

46. Assess his/her work regularly.

**Part Two. Teacher Workplace Buoyancy**

Instruction: Please read the following list carefully. For each statement, select the response that best represents your Agreement or Disagreement. Ranging from 1 (Strongly disagree) to 7 (Strongly agree).

1. I don’t let work stress get on top of me.

2. I think I’m good at dealing with work pressures.

3. I don’t let a bad performance or outcome at work affect my confidence.

4. I’m good at dealing with setbacks at work (e.g., poor performance, negative feedback).

**Part Three. Teacher Cognitive Flexibility**

Instruction: Please read the following list carefully. For each statement, select the response that best represents your Agreement or Disagreement. Ranging from 1 (Strongly disagree) to 6 (Strongly agree).

1. I can communicate an idea in many different ways.

2. I avoid new and unusual situations.

3. I feel like I never get to make decisions.

4. I can find workable solutions to seemingly unsolvable problems.

5. I seldom have choices when deciding how to behave.

6. I am willing to work at creative solutions to problems.

7. In any given situation, I am able to act appropriately.

8. My behavior is a result of conscious decisions that I make.

9. I have many possible ways of behaving in any given situation.

10. I have difficulty using my knowledge on a given topic in real life situations.

11. I am willing to listen and consider alternatives for handling a problem.

12. I have the self-confidence necessary to try different ways of behaving.
